# Right frontal HD-tDCS reveals causal involvement of time perception networks in temporal processing of concepts

**DOI:** 10.1038/s41598-023-43416-z

**Published:** 2023-10-03

**Authors:** Karim Johari, Fatemeh Tabari, Rutvik H. Desai

**Affiliations:** 1 Human Neurophysiology and Neuromodulation Laboratory, Department of Communication Science and Disorders, Louisiana State University, 86 Hatcher Hall, Field House Drive, Baton Rouge, LA 70803 USA; 2 Department of Psychology, University of South Carolina, Columbia, SC USA; 3 Institute for Mind and Brain, University of South Carolina, Columbia, SC USA

**Keywords:** Language, Neurophysiology, Neuroscience, Cognitive neuroscience

## Abstract

Evidence suggests that perceptual and action related features of concepts are grounded in the corresponding sensory-motor networks in the human brain. However, less is known about temporal features of event concepts (e.g., *a lecture*) and whether they are grounded in time perception networks. We examined this question by stimulating the right dorsolateral prefrontal cortex (rDLPFC)—a part of time perception network—using HD-tDCS and subsequently recording EEG while participants performed semantic and time perception tasks. Semantic tasks were composed of event noun duration judgment (EDur), object noun size judgement (OSize), event (EVal) and object noun valence judgement. In the time perception task, participants judged the durations of pure tones. Results showed that cathodal stimulation accelerated responses for time perception task and decreased the magnitude of global field power (GFP) compared to sham stimulation. Semantic tasks results revealed that cathodal, but not sham, stimulation significantly decreased GFP for EDur relative to OSize, and to EVal. These findings provide first causal evidence that temporal features of event words are grounded in the rDLPFC as part of the temporal cognition network and shed light on the conceptual processing of time.

## Introduction

Converging evidence suggests that semantic features of concepts related to action and perception are grounded in the corresponding sensory-motor systems^1–6^. For example, the processing of action-related words (e.g., running) activates primary and higher-order motor areas^2,7–9^. Additionally, movement disorders that primarily affect the motor system (e.g., Parkinson’s disease or stroke) impair action-related words compared to non-action words^10–12^. Similarly, sensory features of concepts also activate corresponding sensory areas in the brain^13–15^. However, less is known about how temporal features of concepts are processed, even though timing is a common semantic constituent of verbs (e.g., *to nap*) and nouns (e.g., *the meeting*). We are aware of two previous studies that suggested that temporal features of event concepts recruit the time perception network, in a manner that is analogous to involvement of sensory-motor systems in processing of sensory and action features of concepts. In the first study, Lai and Desai^16^ found that processing of temporal metaphors *(*e.g., *The hours crawled until the release of the news)* recruited the brain areas that were hypothesized to be involved in time perception. In the second study^17^, we found that N400 event-related potentials (ERPs) were significantly different when participants were asked to judge the duration of event nouns compared to the size judgement of object nouns. This effect was observed over right parietal electrodes, and is reported to be an EEG marker of time perception^18^. Based on these findings, we proposed that temporal features of concepts may be subserved by time perception networks.

In the present study, we sought to provide causal evidence to further bolster this hypothesis. Here, we focused on the involvement of the right dorsolateral prefrontal cortex (rDLPFC) in the conceptual processing of event vs. object nouns. The choice of rDLPFC was based on previous studies that have implicated it in a wide range of time perception tasks such as duration judgement, interval reproduction, and temporal bisection^19–23^. Neuroimaging studies have demonstrated the activation of rDLPFC in duration judgement and interval reproduction tasks^24–26^. Moreover, non-invasive stimulation of rDLPFC temporarily compromises neurotypical adults’ performance in time perception tasks^22,23,27^. Given the role of rDLPFC in time perception, we hypothesized that conceptual processing of event words is partially grounded in the rDLPFC. Here, we tested this hypothesis by stimulating rDLPFC using high-definition transcranial direct current stimulation (HD-tDCS), and subsequently recording EEG data while participants performed time perception and semantic judgement of event and object nouns. HD-tDCS provides higher spatial resolution compared to conventional tDCS by utilizing several small electrodes, which allows delivery of maximum intensity at a pre-defined target^9,28–30^. Moreover, the post stimulation effect of HD-tDCS lasts more than 2 h^31^, which offers sufficient time to complete several tasks in a single session. We examined the effect of stimulation over rDLPFC on the global field power (GFP) of EEG activity associated with event noun duration judgement and time perception tasks. Additionally, object noun size judgement and event and object noun emotional valence judgment tasks were used as control conditions to account for processes related to general task demands and lexical processing. We hypothesized that HD-tDCS over rDLPFC would significantly modulate the RT for time perception task and affect neural correlates of temporal processing within the P300 time window (200–300 ms) relative to sham HD-tDCS. The expected modulation within the P300 time window was based on previous studies showing that P300 ERPs are neural markers of temporal processing^32,33^. Additionally, given the role of rDLPFC in the time perception, cathodal stimulation of rDLPFC would modulate GFP compared to sham stimulation. Based on our recent study^17^, we expected that GFP modulations would occur within the time window of N400 (300–500 ms)^17^ for event duration judgment, but not for control tasks.

## Methods

### Participants

Twenty-three right-handed neurotypical adults-from a large pool of students at Louisiana State University- (Age range: 19–24 years; mean age = 20.23 years; 6 Males) were enrolled in this study. Two participants were excluded due to the absence in their second session leaving 21 who completed two sessions. Power analysis using G*Power software^34^ confirmed that our sample size was sufficient to reach the power of > % 80. All participants were native English speakers with no speech and language impairments and had normal hearing and vision. Participants also did not report any neurological or psychiatric conditions. The study was approved by the Institutional Review Board at the Louisiana State University and was in accordance with the principles outlined in the Declaration of Helsinki. Participants provided a written informed consent, and either received course credits or were monetarily compensated for their participation.

### Materials and experimental design

#### Semantic tasks

Experiment included four blocks of semantic judgment tasks: event noun duration judgment (EDur), object noun size judgment (OSize), event noun valence judgment (EVal), and object noun valence judgment (OVal). While the event nouns represented events with varying duration (e.g., the hike, the cruise, the concert), object nouns represented objects or entities with varying sizes (e.g., the particle, the bazaar, the institute). The stimuli were taken from a previous study^17^ and were selected based on a norming study. Event and object nouns were matched on several psycholinguistic variables, including frequency, length, concreteness, and imageability (Table 1; for the details of the norming study and the full list of stimuli, see Johari et al.^17^).

**Table 1:** Psycholinguistic properties of the event and object nouns.

Characteristic	Event nouns	Object nouns	*p* value
	(n = 160)	(n = 160)	
Log frequency	7.92 ± 1.90	8.05 ± 1.93	0.56^a^
# of letters	6.27 ± 1.95	6.12 ± 1.99	0.50^a^
# of syllables	1.88 ± 0.80	1.89 ± 0.99	0.90^b^
Concreteness	4.05 ± 0.48	4.09 ± 0.42	0.40^a^
Imageability	5.12 ± 0.72	5.11 ± 0.83	0.89^a^
Semantic diversity	1.50 ± 0.25	1.50 ± 0.32	0.98^b^
Valence	5.24 ± 1.60	5.36 ± 1.06	0.43^b^
# of nouns also classified as verbs	73 (45.6%)	73 (45.6%)	1.00^c^
Verb frequency	0.67 ± 0.58	0.64 ± 0.55	0.71^b^
Noun frequency	0.87 ± 0.70	0.91 ± 0.65	0.60^b^

All values are mean ± SD or number of occurrences (%).

^a^T-Test equal variances assumed.

^b^T-Test equal variances not assumed.

^c^Chi-Square test for Independence.

The semantic tasks were comprised of 4 blocks with 40 trials for each block, and it took approximately 25 min to complete all semantic tasks.

**Figure 1:**
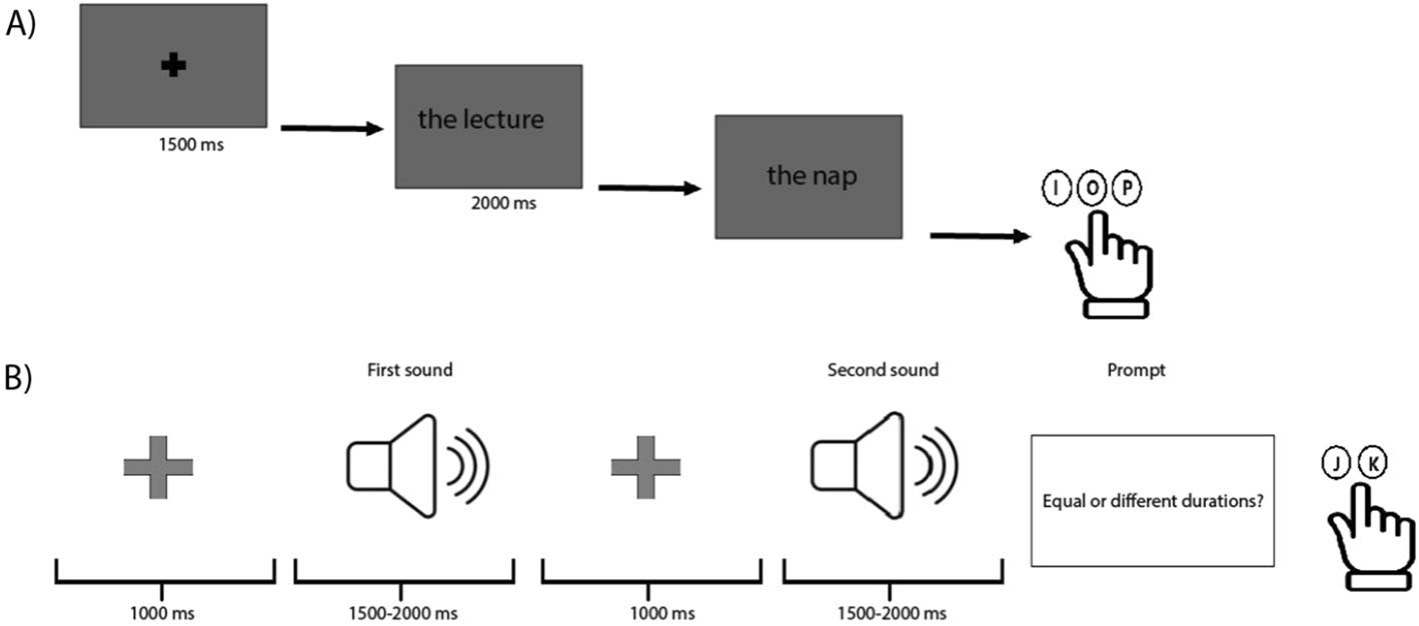
Experimental paradigms for (**A**) semantic task and (**B**) time perception task.

Figure 1A illustrates the structure of a trial for EDur, which was identical for all semantic tasks. Trials began with a fixation cross followed by the first word that stayed on the screen for 2 s. Then the second word appeared on the screen and participants were asked to indicate their response as quickly and accurately as possible. For EDur, participants were instructed to make one of three choices: the first event had greater duration, the second event had greater duration, or both had similar duration. For OSize, they made a similar choice for object sizes. For EVal and OVal, participants were asked to make one of three choices about the stimuli pleasantness: the first word was more pleasant, the second word was more pleasant, or both words were similarly pleasant. For all four tasks, participants were instructed to use three keys on keyboard to indicate their responses with their right hand: “I” for the first word, “P” for the second word and “O” if the first and second words were similar.

#### Time perception task

The experimental design for time perception task is depicted in Fig. 1B. Each trial started with a fixation point followed by the first sound. Then a second sound played, and participants were prompted to indicate whether the duration of two sounds were equal or different using “K” and “J” keys on keyboard, respectively. Sounds were pure tones and made in a custom-made script in MATLAB (MathWorks Inc.) with equal or different duration ranging from 1.5 to 2 s. For trials with equal durations, the tone length was same for the first and the second sounds, whereas for different duration, the length differed. The duration of sounds (in seconds) was determined using the following formula in MATLAB: Duration = rand (1) *(0.5) + 1.5. The choice of durations was based on a recent study that demonstrated causal involvement of rDLPFC in the processing of supra-second intervals^23^. It took approximately 20 min to complete the time perception task.

The order of semantic and time perception tasks was randomized across participants but kept consistent within participants for cathodal and sham sessions. Tasks were written in custom-made scripts in Psychtoolbox^35^ in MATLAB. The software controlled the experimental parameters and synchronized the timing of stimuli and participants’ responses with the EEG signals.

### HD-tDCS configurations

**Figure 2:**
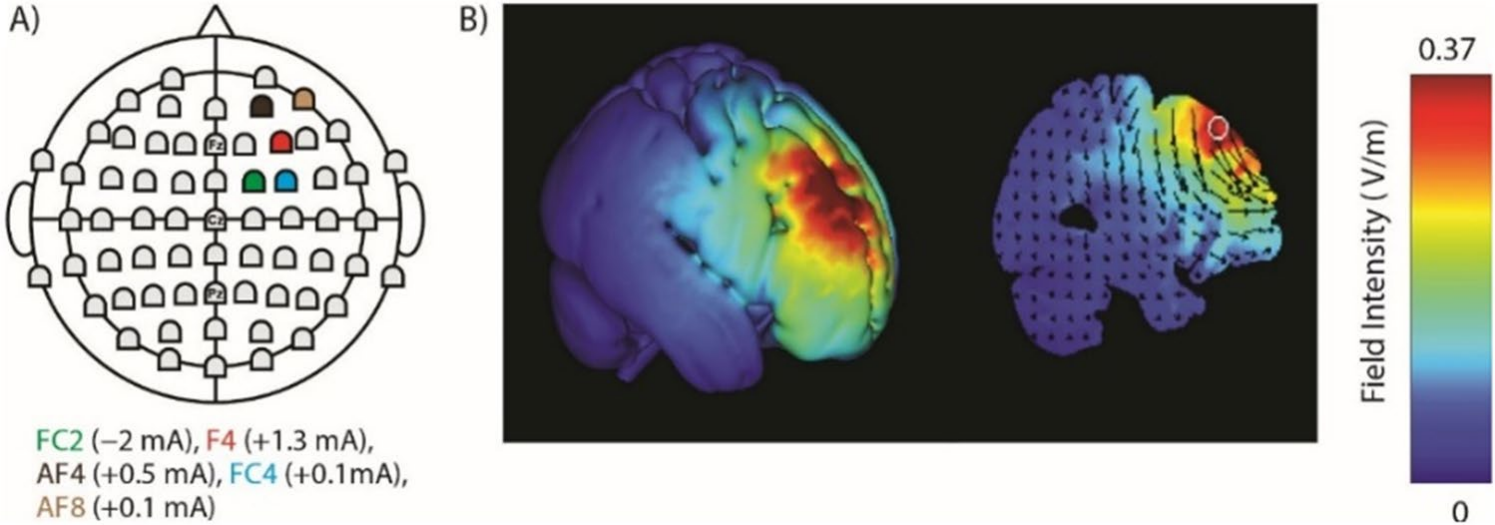
(**A**) The electrode locations and their intensities. (**B**) The modeled current follow based on the electrode configuration in panel (**A**).

HD-explore and HD-target software (Soterix Medical Inc., NY, USA) were used to obtain electrode configurations to induce maximal focality and intensity within rDLPFC. Figure 2A shows the locations of electrodes and their intensities. The modelled current follow is depicted in Fig. 2B.

### Procedure

Participants underwent two counterbalanced sessions in which they received cathodal and sham stimulations with approximately a week apart to minimize carry-over effect. A M×N-9 HD-tES stimulator (Soterix Medical Inc., NY, USA) was used to deliver cathodal and sham stimulations. The electrodes were placed based on 5–10 international montage using an HD cap to ensure that electrodes were secured in place (Soterix Medical Inc., NY, USA). HD-conductive gel was applied to establish a stable connection between electrodes and participants’ scalp. For cathodal stimulation, the modeled electrical current was applied over rDLPFC for 25 min. During 25-min sham condition, the stimulation was delivered only for 60 s at the beginning and 60 s at the end to induce scalp sensation of stimulation. Participants were asked to rate the level of their pain and unpleasantness induced by stimulation. Participants’ rating was recorded 3 times in each session: 30 s and 12.5 min after the onset of stimulation and 30 s before the end of stimulation to ensure that the participants are not experiencing pain but receive stimulation throughout the experiment. They reported their pain and unpleasantness on a scale of 1–10, with 10 being associated with extreme pain or unpleasantness. Participants’ scores were not significantly different for cathodal stimulation compared to sham condition (all *p* > 0.35).

### EEG acquisition

Each stimulation session was followed by an EEG recording wherein participants performed semantic and time perception tasks. EEG data were acquired using a 64-channel actiCHamp Plus EEG system (Brain Products GmbH, Germany). Electrodes were placed on a cap based on 5–10 international montage. We applied conductive gels to each electrode to ensure the impedance is below 5 kΩ. EEG data was recorded using Brainvision recorder software (Brain Products GmbH, Germany), with a sampling rate of 500 Hz and referenced to the Fz electrode. EEG preparation took approximately 20 min.

### Behavioral analysis

For semantic tasks, reaction times (RT) were calculated by obtaining the temporal delay between the onset of the second word and participants’ response. For time perception task, RTs were computed by subtracting the timing between the prompt onset and participants’ responses. For both semantic and time perception tasks, for each participant, we excluded RTs that were 3 standard deviations below or above the mean RT separately for each task and stimulation. For semantic tasks, the cleaned data were submitted to a linear mixed effect model to examine the effect of stimulation, tasks and their interaction on RTs as fixed effects (RT ~ task + stimulation + task*stimulation + (1| participants) + (1|items)). Participants and items were included as random effects. For time perception task, we included stimulation as a fixed effect and participants and items as random effects (RT ~ stimulation + (1| participants) + (1|items)). We also used a logistic mixed effect model (ACC ~ stimulation + (1| participants) + (1|items), family = binomial)) on accuracy rate for time perception task by including stimulation as a fixed effect and participants and items as random effects. Note that the accuracy for semantic tasks is subjective, especially for the valence tasks. Due to its subjective nature, accuracy is not a meaningful measure for these tasks. The purpose of semantic tasks was to direct participants’ attention towards magnitude or valence features of the concepts. Therefore, we did not perform any statistical analysis on accuracy rates. However, participants’ performance were above chance with overall accuracy > 65% if an experimenter-defined ‘correct response’ is used.

### EEG analysis

EEG Data were analysed using EEGLAB software^36^. First, the continuous EEG signal was visually inspected to detect noisy electrodes. The broken and noisy electrodes were interpolated using sphere approach in EEGLAB. Then, the data were re-referenced using common average of all electrodes. High pass filter of 0.5 Hz was applied to the data, and muscle artifacts, line noise and eye blinks were detected and removed by applying Independent Component Analysis (ICA). Then cleaned data was low pass filtered at 30 Hz and epoched from 200 before to 1000 ms after the onset of first sound and first word for time perception and semantic tasks, respectively. For semantic tasks, data were segmented relative to the onset of first word to avoid the overlap between N400 and EEG activity associated with motor planning which would occur around the onset of the second word. Epoched data were baseline normalized to -200 to 0 ms on trial-by-trial basis. We used global field power (GFP) as an unbiased EEG measure to compare our tasks. GFP is a reference independent index that is defined as the magnitude of the electrical activity in the field in any given time across all channels^37,38^. GFP provides a more simplified statistical design and does not have arbitrary or bias-prone selection of channels. For each participant, we calculated GFP by obtaining the standard deviation of amplitudes in epoched data from all 64 electrodes for each data point, separately for each task and stimulation condition. Statistical analyses were performed using permutation-based clustering^39^ with a conservative threshold of *p* < 0.01 and 10,000 permutations to compare the magnitude of GFP between tasks separately for cathodal and sham stimulations, and between cathodal and sham within each task. Permutation-based clustering is a non-parametric test that offers an unbiased approach for the statistical analysis of EEG data compared to classical methods in which analyses are prone to bias due to potential post-hoc selection of time windows.

We computed GFP for all four semantic tasks (EDur, EVal, OSize, OVal) in cathodal and sham stimulations, locked to the onset of the first word. Similarly, GFP locked to the onset of the first sound was obtained for the time perception task in both cathodal and sham stimulations. We contrasted EDur to OSize (comparing magnitude judgments for event and object nouns), and EDur to Eval (comparing duration vs. valence tasks for the same words). As a control contrast, we compared OSize to OVal.

## Results

### Behavioral results

**Figure 3:**
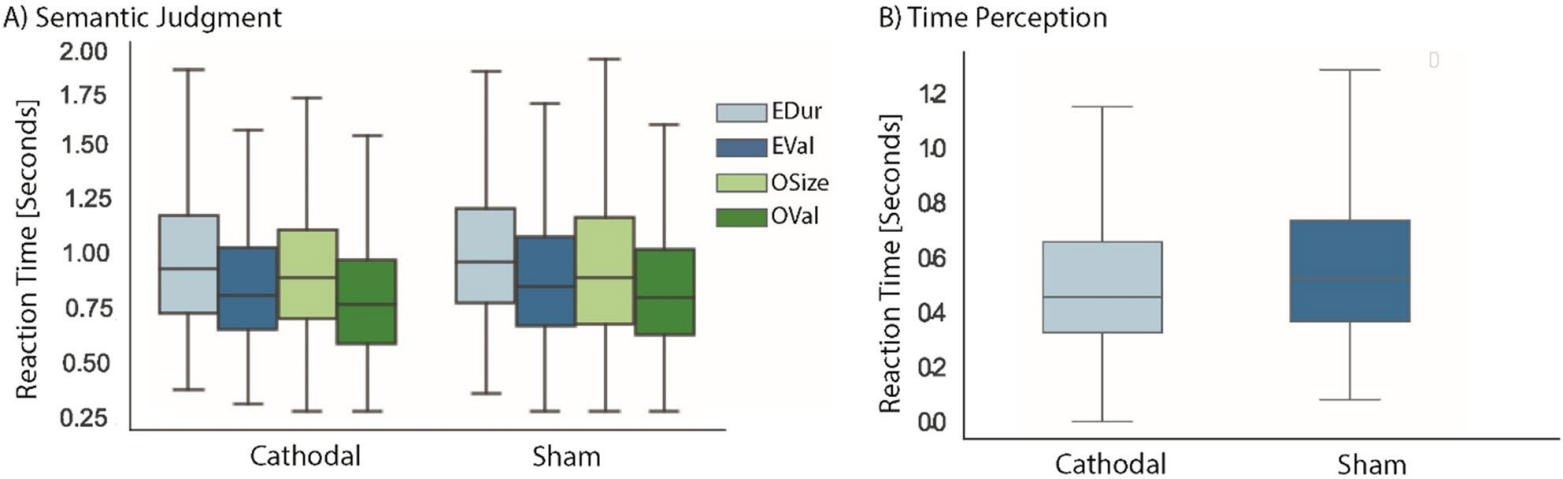
Mean RTs in seconds for **A**) semantic (no significant stimulation effect) and **B**) time perception tasks (a significant stimulation effect) for cathodal and sham HD-tDCS.

Semantic tasks: Fig. 3A shows the RTs for semantic task following cathodal and sham stimulations. A linear mixed effect model did not show a significant effect of stimulation (F (1, 4231) = 0.97, *p* = 0.32). There was a significant effect of task (F (3, 4231) = 45.68, *p* = 0.001), however this effect was qualified by a stimulation and task interaction (F (3, 4223) = 7.40, *p* < 0.0001). Post hoc analysis verified that significant interaction between task and stimulation was not driven by the hypothesized contrasts as revealed by a non-significant interaction between tasks (EDur vs. OSize) and stimulation (cathodal vs. sham) (F (1, 1866) = 2.07, *p* = 0.15). Finally, cathodal stimulation did not significantly modulate RTs relative to sham stimulation for EDur (z = − 2.77, *p* = 0.15) and OSize (z = 2.85, *p* = 0.12).

#### Time perception

The results of a linear mixed effect model revealed cathodal stimulation improved RTs compared to sham HD-tDCS (t(3910) = 7.26, *p* < 0.0001; Fig. 3B). Logistic mixed effect results did not indicate a significant difference between accuracy rates of cathodal and sham stimulations (z = − 1.23, *p* = 0.22; accuracy: sham: 80% (SD: 39%), cathodal: 82% (SD: 40%).

### EEG results

#### Semantic tasks

**Figure 4:**
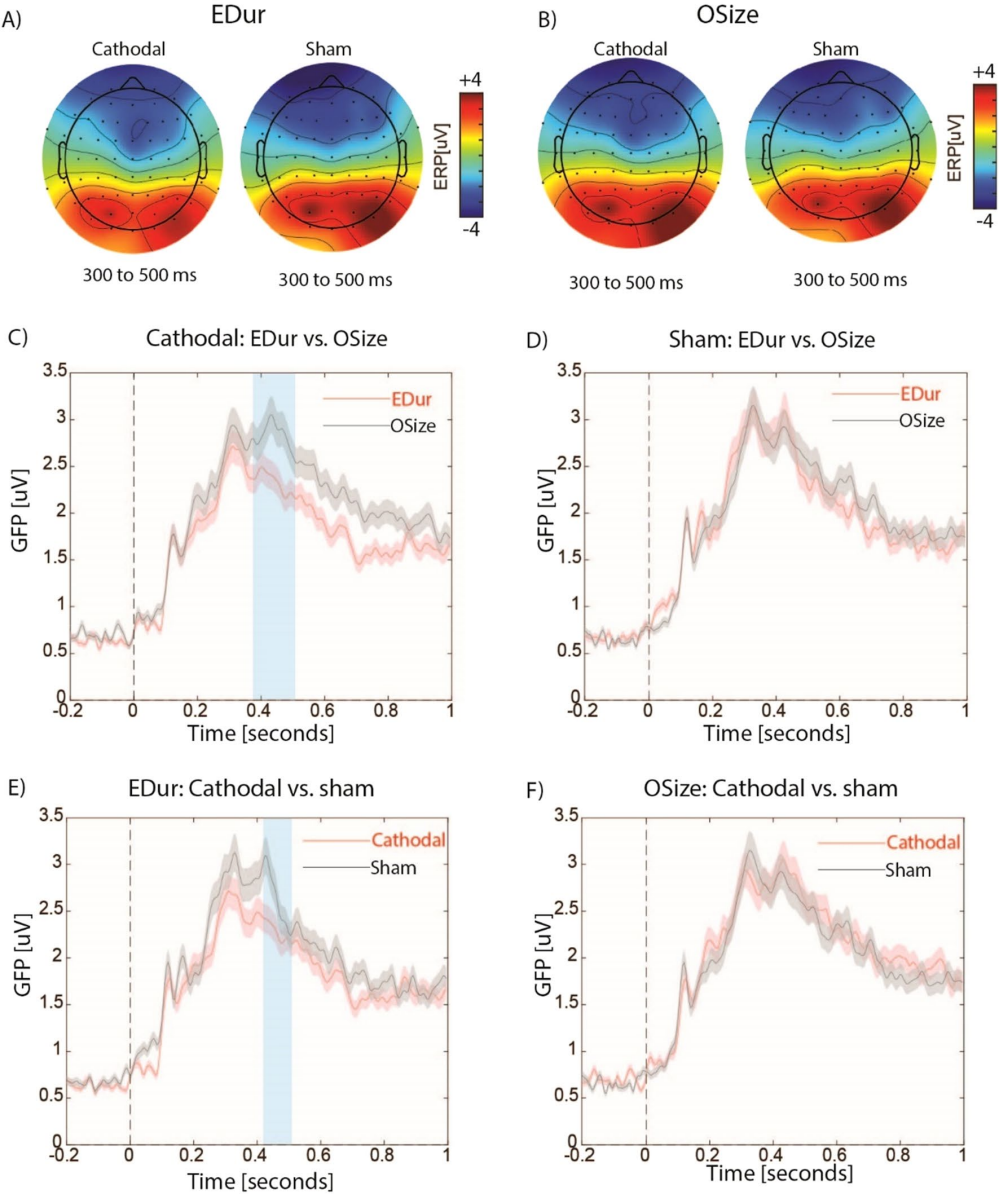
The topographical distribution of ERP amplitudes for all electrodes from 300 to 500 ms after the onset of the first word separately for **A**) EDur and **B**) OSize. Panels **C**) and **D**) illustrate the overlaid temporal profile of GFPs from 200 ms before to 1000 ms after the onset of the first word for EDur vs. OSize following cathodal and sham stimulation respectively. Panels E) and F) compare cathodal vs. sham stimulation for EDur and Osize respectively. Shaded areas in red and black colors show the standard errors of the mean GFP. Blue shaded areas show significant differences at the threshold of *p* < 0.01 within the time window of N400.

**Figure 5:**
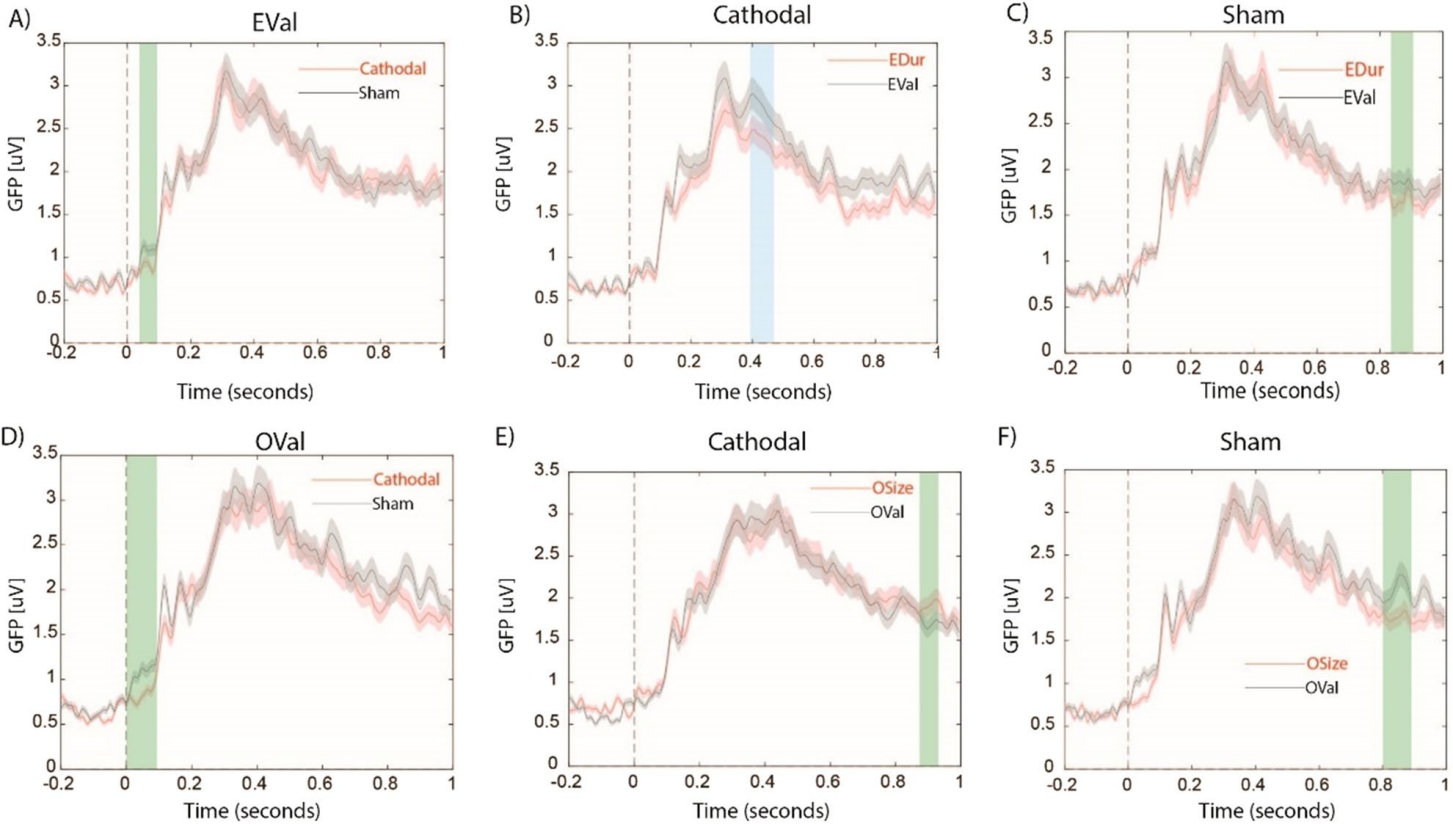
Overlaid temporal profile of GFPs from 200 ms before to 1000 ms after the onset of the first word for **A**) cathodal vs. sham for EVal, **B**) EDur vs. EVal following sham, **C**) EDur vs. EVal following cathodal **D**) cathodal vs. sham for OSize, **E**) OSize vs. OVal following cathodal, and **F**) OSize vs. OVal following sham. Shaded areas in red and black colors show the standard errors of the mean GFP. Blue shaded area shows significant differences at the threshold of *p* < 0.01 within time window of N400. Green shaded area illustrates differences captured by cluster-based permutation analysis that were out of the time window of interest.

EDur and OSize elicited prominent ERP activities over bilateral frontal and right parietal electrodes around the time window of N400 in both cathodal and sham conditions (Fig. 4A and B). Permutation based clustering with *p* < 0.01 showed that cathodal HD-tDCS over rDLPFC significantly decreased the magnitude of GFP for EDur compared to OSize (Fig. 4C). This effect was not observed in sham condition (Fig. 4D). Relative to sham, cathodal stimulation significantly attenuated the magnitude of GFP for EDur (Fig. 4E), but not OSize (Fig. 4F), around the time window of N400. We also observed a significant decrease in the magnitude GFP for EDur relative to EVal around the time window of N400 following cathodal (Fig. 5B) but not sham (Fig. 5C) HD-tDCS. Cathodal stimulation did not result in a significant difference within time window of N400 either at the threshold of *p* < 0.01 or at *p* < 0.05 for OSize vs. OVal (Fig. 5E). GFP magnitude did not significantly differ for EDur vs. EVal (Fig. 5C) and OSize vs. OVal (Fig. 5F) following sham HD-tDCS. Finally, cathodal HD-tDCS significantly decreased the GFP for EVal as well as OVal within 100 ms after the onset of the first word compared to sham stimulation (Fig. 5A and D). However, they were not significantly different in the time window of N400.

#### Time perception

**Figure 6:**
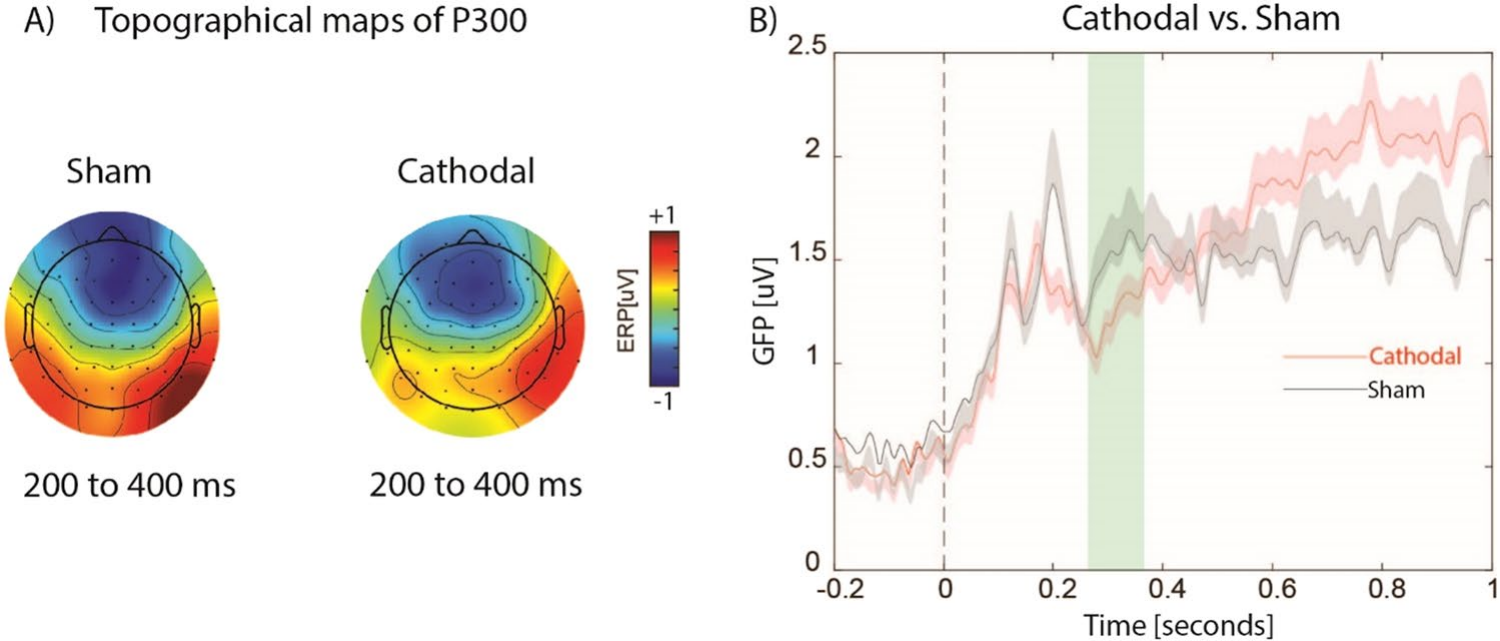
(**A**) the topographical distribution maps of ERPs for all electrodes from 200 to 400 ms after the onset of the first sound for cathodal and sham stimulations. (**B**) the temporal profile of GFP from 200 before, to 1000 ms after the onset of the first sound for cathodal and sham stimulations. Shaded areas in red and black colors show the standard errors of the mean GFP. Green shaded area shows significant differences at the threshold of *p* < 0.01.

Temporal judgement task elicited bilateral prefrontal, frontocentral and right parietal activity at time window of 200–400 ms for both cathodal and sham stimulations (see Fig. 6A). Cluster-based permutation analysis at the threshold of *p* < 0.01 revealed that cathodal stimulation attenuated the magnitude of GFP compared to sham around the time window of P300 (Fig. 6B).

## Discussion

We examined the causal role of rDLPFC in processing of temporal features of concepts in event nouns. To test the selectivity of this effect, two control tasks were used: size judgments on closely matched object nouns, and valence judgments on event nouns. The GFP magnitude was comparable for EDur and OSize following sham stimulation, suggesting that both magnitude related features depend on shared neural network. However, after stimulating the rDLPFC, the decrease in the magnitude of GFP was evident only for EDur and not for OSize, indicating that this region selectively supports temporal features of event nouns. This effect is unlikely to be explained by differences between event and object nouns, as both were closely matched on several psycholinguistic properties, including frequency, concreteness, and imageability. The two tasks also had comparable difficulty and executive demands, as measured by RTs. This view is further supported by the findings in the time perception task that cathodal stimulation attenuated the magnitude of GFP compared to sham stimulation at time window 200–300 ms after the first sound. This time window falls within the P300 ERPs which is hypothesized to support temporal processing^32,40^.

The second control task, EVal represents an even stronger control condition for EDur, which used an identical set of words (across participants), and only attention was manipulated by directing it to a different set of features. A reduction in GFP was observed for EDur relative to EVal after cathodal, but not following sham stimulation. This provides further evidence that this effect is specific to the duration judgment of event nouns, and is not a general effect related to processing of words or to performing a task. The rDLPFC is implicated in executive control and decision making^41,42^, which were required for the time perception and semantic tasks. The specificity of the effects suggests a role of rDLPFC beyond that in domain general processes, in temporal processing. The confounding effects related to providing a motor response (button-press) and decision-making are also minimized by analyzing ERPs time-locked to the onset of first stimulus.

Our EEG results did not directly translate into behavioral changes as measured by RTs in semantic tasks, particularly for EDur vs. OSize. RTs are influenced not just by semantics of the individual words, but also by control and executive processes required to compare typical instances of each event or object and related features, such as decision-making. Hence, we avoided time-locking to the second word, and instead focused the GFP analysis on the first word.

A distributed set of regions and networks are implicated in time perception and estimation^26,43,44^. There is no evidence for dedicated timing machinery, or a contiguous set of regions responsible for timing. A meta-analyses of neuroimaging studies by Wiener et al.^26^ and Cona et al.^45^ identified an extensive set of regions for estimation of time intervals ranging from 200 ms to 24 s, including the rDLPFC, SMA, right inferior parietal lobule (IPL), supramarginal gyrus (SMG), and other perisylvian areas. Given the distributed nature of this circuit, an approach that uses GFP is suitable. While we stimulated one node of this circuit (rDLPFC), it is certainly possible and likely that other parts of the circuit also play important roles. For example, repetitive transcranial magnetic stimulation (rTMS) of the rIPL causes altered temporal perception^46–48^. Whether this stimulation would also translate to effects on semantic judgments of duration remains a question for future work.

Similar GFP magnitudes for EDur and OSize during sham stimulation can be argued to be consistent with “A Theory Of Magnitude” (ATOM) account^49,50^, which hypothesizes a shared magnitude system subserving domains such as time, space and quantity. Indeed, several behavioral and neuroimaging studies have supported the notion of a common magnitude system that is used for space, time, number, and actions^51–54^. However, the selective modulation of GFP for EDur, centered on frontal and right parietal electrodes, also suggests differences between temporal and spatial judgments. Based on a meta-analysis, Cona et al.^45^ suggested space–time gradients, rather than a completely overlapping system, in bilateral frontal (both lateral and superior medial) as well as right parietal areas. This suggestion is consistent with the current results, if our chosen stimulation location is assumed to be closer to the temporal end of the gradient. Our findings are also consistent with the suggestion that the mapping between space and time is not symmetrical (as would be predicted by ATOM)^55–57^. A common magnitude system may exist only in early childhood, which is gradually differentiated to handle space, time, and number separately^58,59^. Here, the similarity between responses to temporal and spatial semantic tasks to that of the respective temporal and spatial systems indicates grounding of the semantic processing of these features that appears to mirror the underlying systems that are at least partially separated.

The GFP for the time perception task was modulated in the 200–300 ms window, whereas GFP changes occurred around 400 ms for semantic tasks. We do not expect identical time course for temporal judgments on pure tones and semantic judgments on words, since the processing of visual word forms and subsequent activation of word meaning is a complex process involving a number of stages. After low-level visual analysis, orthographic analysis of the word form takes place at sublexical and lexical levels. This leads to spreading of activation to the semantic system and relevant concept features, depending on the salience of features and top-down factors such as task demands and context. The duration feature of pure tones can be accessed from acoustic analysis and this is expected to be more rapid. The critical finding is that attenuation of GFP magnitude was caused by stimulation of rDLPFC for both temporal and semantic duration judgments selectively. This supports the proposal that temporal features of concepts are subserved by time perception network particularly within rDLPFC.

Finally, the neuromodulatory effect of sham stimulation in tDCS as a control condition is not fully understood. Some evidence suggests that sham stimulation, even for a short period of time (< 10 s), can induce neural and behavioral changes like actual stimulation^60–62^. Therefore, it is plausible that sham stimulation in the present study induced some changes in cortical excitability, however this did not yield to meaningful differences in GFP between duration and size tasks.

## Conclusion

We provided first evidence that stimulation of rDLPFC—a brain region hypothesized to subserve temporal cognition—causes attenuation of GFP of EEG signals for a time perception task, and selectively attenuated the GPF for event noun duration judgments. This suggests that temporal features of concepts are grounded in time perception circuits. This meshes with and extends the body of findings that show similar grounding of visual, auditory, action, and emotion features of concepts. Future neuroimaging and neurostimulation studies are warranted to examine the role of other brain regions involved in time perception and examining their relationship with spatial perception.

## Data Availability

The data would be available upon reasonable request by the corresponding author.
